# Supplementary material for: Active site specificity profiling datasets of matrix metalloproteinases (MMPs) 1, 2, 3, 7, 8, 9, 12, 13 and 14
Source: Data Brief. 2016 Feb 22;7:299–310. doi: 10.1016/j.dib.2016.02.036 (PMC4777984; doi:10.1016/j.dib.2016.02.036)
Supplement: Supplementary file 10 — Supplementary material [file mmc10.zip › WebPICS_hMMP3_T_1%/P1.html]

 

PICS results


|  |  |
| --- | --- |
| **P1\_A**  23 in 199 sites   11.6 %    effects > 10 perc. pnts.  (vice-versa in brackets)  P3prime\_A: 13.5 (12.4) |  |
| **P1\_D**  21 in 199 sites   10.6 %    effects > 10 perc. pnts.  (vice-versa in brackets)  P3\_I: 15.3 (18.8)   P1prime\_F: 10.3 (26.9) |  |
| **P1\_E**  40 in 199 sites   20.1 %    effects > 10 perc. pnts.  (vice-versa in brackets)  P3\_L: 18.4 (32.1) |  |
| **P1\_F**  8 in 199 sites   4.0 %    effects > 10 perc. pnts.  (vice-versa in brackets)  P2\_M: 34.0 (38.9)   P1prime\_V: 46.9 (12.1)   P2prime\_M: 34.0 (38.9) |  |
| **P1\_G**  18 in 199 sites   9.0 %    effects > 10 perc. pnts.  (vice-versa in brackets)  P3\_V: 27.8 (22.8)   P1prime\_Q: 11.2 (18.3)   P1prime\_W: 19.2 (57.7) |  |
| **P1\_N**  24 in 199 sites   12.1 %    effects > 10 perc. pnts.  (vice-versa in brackets)  P3\_A: 12.9 (12.9)   P2prime\_V: 16.1 (14.8) |  |
| **P1\_P**  18 in 199 sites   9.0 %    effects > 10 perc. pnts.  (vice-versa in brackets)  P2\_F: 12.2 (24.3)   P1prime\_V: 17.7 (10.4)   P3prime\_H: 19.2 (57.7) |  |
| **P1\_Q**  11 in 199 sites   5.5 %    effects > 10 perc. pnts.  (vice-versa in brackets)  P3\_H: 15.2 (27.8)   P2prime\_I: 25.3 (12.7)   P3prime\_T: 29.4 (23.1) |  |
| **P1\_S**  16 in 199 sites   8.0 %    effects > 10 perc. pnts.  (vice-versa in brackets)  P3\_V: 13.9 (10.2)   P2\_Q: 11.3 (12.0)   P2\_S: 24.2 (27.7)   P3prime\_C: 11.0 (58.7)   P3prime\_E: 18.0 (20.6) |  |
